# Metabolic Phenotype and Microbiome of Infants Fed Formula Containing *Lactobacillus paracasei* Strain F-19

**DOI:** 10.3389/fped.2022.856951

**Published:** 2022-04-26

**Authors:** Hanna Lee, Zailing Li, Britt Christensen, Yongmei Peng, Xiaonan Li, Olle Hernell, Bo Lönnerdal, Carolyn M. Slupsky

**Affiliations:** ^1^Department of Food Science and Technology, University of California, Davis, Davis, CA, United States; ^2^Department of Pediatrics, Peking University Third Hospital, Beijing, China; ^3^Arla Foods amba, Arla Innovation Center, Skejby, Denmark; ^4^Department of Child Health Care, Children's Hospital, Fudan University, Shanghai, China; ^5^Department of Child Health Care, Children's Hospital of Nanjing Medical University, Nanjing, China; ^6^Department of Clinical Sciences, Pediatrics, Umeå University, Umeå, Sweden; ^7^Department of Nutrition, University of California, Davis, Davis, CA, United States

**Keywords:** gut microbiota, metabolome, infant formula, *Lactobacillus*, probiotics

## Abstract

Early childhood nutrition drives the development of the gut microbiota. In contrast to breastfeeding, feeding infant formula has been shown to impact both the gut microbiota and the serum metabolome toward a more unfavorable state. It is thought that probiotics may alter the gut microbiota and hence create a more favorable metabolic outcome. To investigate the impact of supplementation with *Lactobacillus paracasei* spp. *paracasei* strain F-19 on the intestinal microbiota and the serum metabolome, infants were fed a formula containing *L. paracasei* F19 (F19) and compared to a cohort of infants fed the same standard formula without the probiotic (SF) and a breast-fed reference group (BF). The microbiome, as well as serum metabolome, were compared amongst groups. Consumption of *L. paracasei* F19 resulted in lower community diversity of the gut microbiome relative to the SF group that made it more similar to the BF group at the end of the intervention (4 months). It also significantly increased lactobacilli and tended to increase bifidobacteria, also making it more similar to the BF group. The dominant genus in the microbiome of all infants was *Bifidobacterium* throughout the intervention, which was maintained at 12 months. Although the serum metabolome of the F19 group was more similar to the group receiving the SF than the BF group, increases in serum TCA cycle intermediates and decreases in several amino acids in the metabolome of the F19 group were observed, which resulted in a metabolome that trended toward the BF group. Overall, *L. paracasei* F19 supplementation did not override the impact of formula-feeding but did impact the microbiome and the serum metabolome in a way that may mitigate some unfavorable metabolic impacts of formula-feeding.

## Introduction

It is well established that early childhood nutrition can impact long-term health. Development of the gut microbiome is important for ensuring proper gut function and development of the immune system (1), as well as development of other organs including the brain (2). There are many factors that influence the development of the microbiome, including infant diet. It has been shown that formula-fed (FF) and breast-fed (BF) infants harbor distinct microbiomes (3–8), with BF infants having a microbiome dominated by bifidobacteria and lactobacilli. Formula-feeding has been linked with metabolic stress that includes metabolic and immune alterations such as higher serum insulin coupled with higher serum amino acids, altered cytokines and blood lipids (5, 7, 9–11), as well as higher infection rates during the first year of life (12) compared with BF infants. Furthermore, FF infants are more likely to develop obesity and metabolic dysfunction later in life than BF infants (13, 14).

Probiotics are microorganisms thought to confer a health benefit when consumed through altering the composition of the intestinal microbiota. For premature infants, they are becoming more accepted as prophylaxis against necrotizing enterocolitis (15, 16). Indeed, evidence has shown that provision of probiotics, such as *Bifidobacterium animalis lactis, B. bifidum, B. infantis, Lactobacillus acidophilus, L. reuteri*, and *L. rhamnosus*, in preterm and term infants as well as rhesus monkey infants has a significant impact on the microbial community structure (4, 17–19). Probiotic effects are also population-specific due to differences in the basic commensal bacteria and environment (20). Among probiotic strains, *L. paracasei* subsp. *paracasei* F19 (F19) is a GRAS-approved (GRN No. 840) strain that exhibits genetic stability throughout production (21). F19 survives gastric transit in infants (22), and actively interacts with the gut epithelium and immune system while exhibiting antioxidative and proteolytic activities in the gut (23). Its main glucose fermentation product is lactic acid, a metabolite that is known to have antimicrobial, immune-modulating, and intestinotrophic effects (24). Clinically, research on F19 has largely focused on gastrointestinal health (25–27) and immune modulation (28), and to a lesser extent on protection against obesity (23).

We recently conducted a trial to investigate the impact of feeding an infant formula containing a probiotic strain, *Lactobacillus paracasei* spp. *paracasei* strain F-19 (F19) in infants from 21 days (± 7 days) until 4 months of age (29). Overall, the F-19 supplemented formula was well-tolerated, with few adverse effects (29). To understand the impact of supplementation with F-19 more fully, we report on the serum metabolome and fecal microbiome in the same cohort of infants.

## Results

### Study Participants

Demographic data on study participants are shown in Table 1. Two sets of subjects were randomly chosen for serum metabolomics analysis at 4 months and microbiome analysis at three timepoints: baseline, 4 months, and 12 months (with some subjects overlapping in the two sets). Approximately equal numbers of male and female subjects comprised the breast-fed reference cohort (BF) and the cohort provided the *Lactobacillus paracasei* spp. *paracasei* strain F-19 probiotic (F19); however, there were more female than male subjects in the standard formula group (SF) in our dataset. Additionally, a greater number of infants were delivered vaginally in the BF group compared to the F19 or SF groups, and the F19 group had a greater number of vaginally born infants compared to the SF group. Mean birthweight was not significantly different amongst the groups. We previously reported that both formula groups experienced similar infectious episodes during the intervention, but only the SF group had significantly more days and episodes of fever than the BF group (29). For these subsets of infants, we did not find any difference in the frequency of antibiotic use or diarrheal episodes among groups. We observed significantly higher serum IFN-γ in the SF group compared to the BF and F19 groups, which is what was published previously for this cohort (30). For the subset of infants used for metabolomics, we also observed higher serum IL-2 (general T cell stimulation) in the F19 group compared to the BF group, but this was not significant for the subset of infants used for microbiota analysis (data not shown).

**Table 1:** Characteristics of infants used for analysis of the serum metabolome and the fecal microbiome.

**Variable**	**Group**	**BF**	**F19**	**SF**	***P*-value _**BF vs F19 vs SF**_**
Total *n*	Metabolome	42	41	40	-
	Microbiome	37	43	41	-
Region (B/N/S)	Metabolome	13/17/12	9/16/16	8/14/18	0.590
	Microbiome	11/17/9	7/19/17	9/15/17	0.403
Sex %F	Metabolome	52^a^	48^a^	65^b^	0.035*
	Microbiome	51^a^	51^a^	68^b^	0.018*
Delivery mode % Vaginal	Metabolome	67^a^	49^b^	38^b^	<0.001*
	Microbiome	65^a^	53^a^	32^b^	<0.001*
Birthweight (mean ± SEM)	Metabolome	3,322 ± 50	3,397 ± 65	3,276 ± 56	0.487
	Microbiome	3,313 ± 44	3,416 ± 58	3,253 ± 55	0.397
Antibiotic use (1–4 months)	Metabolome	2	4	7	0.157
	Microbiome	Excluded	Excluded	Excluded	-
Antibiotic use (4–8 months)	Metabolome	7	14	12	0.160
	Microbiome	5	14	13	0.103
Diarrhea (1–4 months)	Metabolome	3	7	1	0.057
	Microbiome	0	5	1	0.056
Diarrhea (4–8 months)	Metabolome	4	7	9	0.286
	Microbiome	2	5	4	0.682

Significance was tested by FDR-corrected one-way ANOVA of log-transformed values for the birth weight (g) and by Pearson's Chi-squared test with simulated p-value (based on 2,000 replicates to increase the sample size) for all the other variables. When significance was found (*p<*0.05), pair-wise comparisons were performed*.

*Region: B, Beijing; N, Nanjing; S, Shanghai*.

*Superscript letters indicate significance differences between groups (FDR *p* < 0.05)*.

### Impact of F19 on the Fecal Microbiome

**Figure 1:**
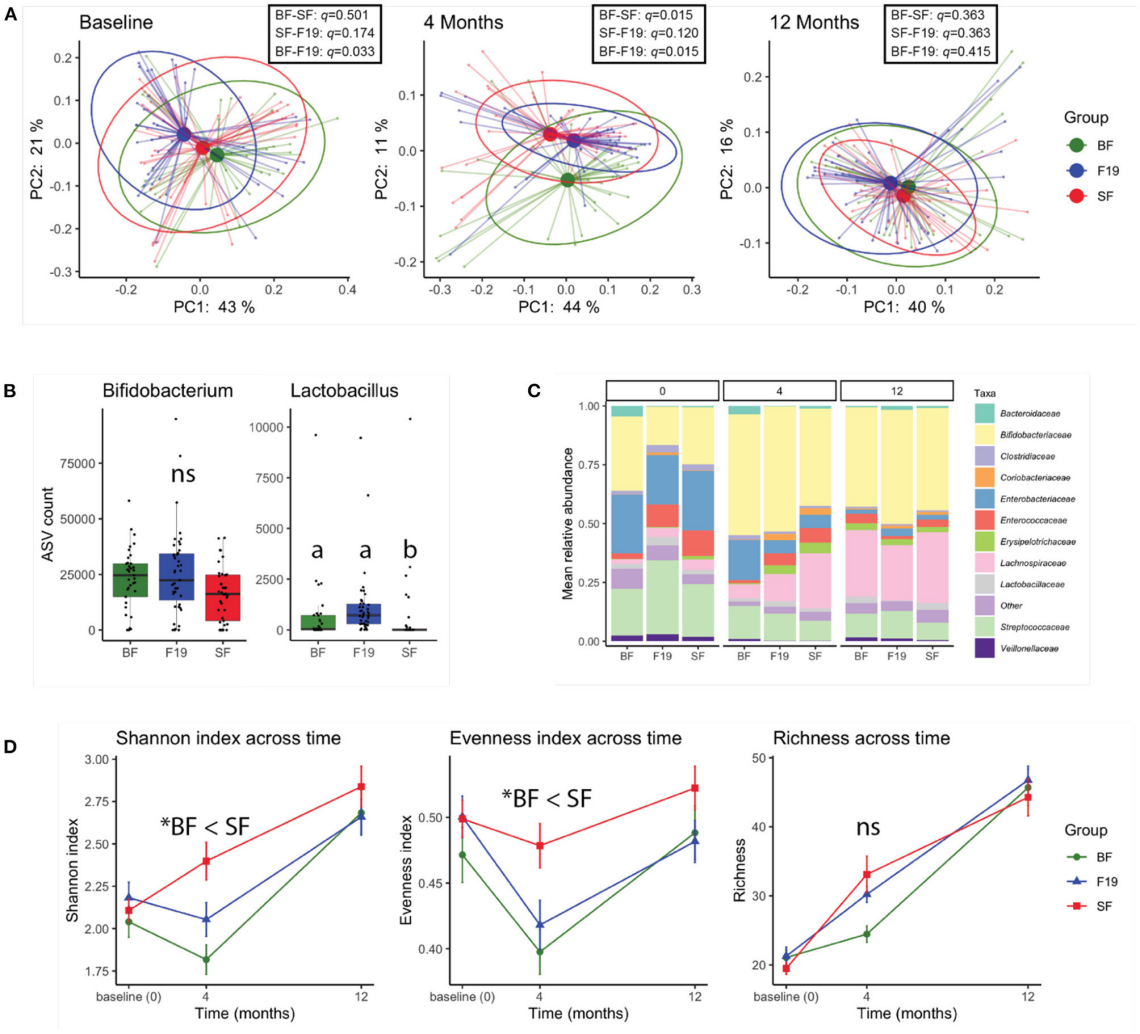
16S rRNA analysis reveals a moderate effect of *L. paracasei* F19 supplementation. **(A)** Principal coordinates analysis (PCoA) of fecal microbiota at baseline (prior to the intervention), 4 months (at the end of the intervention), and 12 months based on the weighted UniFrac distance metric. Results of Pairwise PERMANOVA are provided in the table insert. **(B)** Amplicon sequence variant (ASV) counts of *Bifidobacterium* and *Lactobacillus* genera for each group at 4 months of age. The different letters indicate statistical significance (ANCOM FDR < 0.05). **(C)** Bar plots of the mean relative abundance at family level of taxonomy in each group at baseline (0), 4, and 12 months of age. Unclassified family or the family with < 2.5% relative abundance were grouped as *Other*. **(D)** Fecal microbial alpha-diversity represented as the Shannon, Evenness, and Richness indices over time (*pairwise Kruskal-Wallis *q* < 0.05). Values are shown as mean ± SE. In all panels, the *L. paracasei* F19 supplemented group is represented in blue, the standard formula group in red, and the breastfed reference group in green. A total of 121 samples (*n* = 37 BF, *n* = 41 SF, *n* = 43 F19) were analyzed.

Based on the weighted UniFrac distance matrix, principal coordinates analysis (PCoA) revealed that at baseline (3 wk ± 7 d), the infants assigned to the F19 group were significantly different from the BF group (pairwise PERMANOVA *q* = 0.033; *n* = 999 permutations) but were not different from the SF group (*q* = 0.174). At the end of the intervention, when infants were 4 months of age, the two formula groups were not different and together were significantly different from the BF group (pairwise PERMANOVA *q* = 0.015 (BF-SF), 0.015 (BF-F19); *n* = 999 permutations). By 12 months of age, fecal microbiota was indistinguishable between all three groups (Figure 1A).

Differential abundance analysis revealed that at 4 months, the feces of infants in the F19 group were enriched in members of the *Lactobacillus* genus compared to the SF group (ANCOM FDR < 0.05, tested on relative abundance), and were not significantly different from the BF group (Figure 1B). Aside from higher relative abundance of *Lactobacillus*, no differences at the genus level were observed in the F19 group relative to the SF group. Interestingly, the relative abundance of *Bifidobacterium* was not significantly different between the groups (Figure 1B). Aside from higher *Lactobacillaceae* in the F19 group compared to the SF group at 4 months, the relative abundances of bacterial families were comparable between the formula groups. Both formula groups were different from the BF reference group with respect to the relative abundance of several bacterial families including *Lachnospiraceae, Veillonellaceae, Enterobacteriaceae, Erysipelotrichaceae*, and *Enterococcaceae* (Figure 1C). Notably, group differences were no longer apparent at 12 months, as the relative abundance of bacterial families including *Lactobacillaceae* were similar in all three groups (ANCOM FDR>0.05). This showed that increased *Lactobacillus* through F19 supplementation did not persist 8 months after the end of intervention. Throughout the study, *Bifidobacterium* was a dominant genus in all groups. Analysis of the diversity of the microbiome revealed that BF infants had significantly lower Shannon diversity at all timepoints compared to the SF group, with lower evenness at all three timepoints, and lower growth of richness from baseline to 4 months (Figure 1D). The F19 group tended to have lower Shannon diversity than the SF group at 4 and 12 months, which was reflected as lower evenness at both timepoints, and was not significantly different from the BF group at either time point.

### Impact of F19 Supplementation on the Serum Metabolome

**Figure 2:**
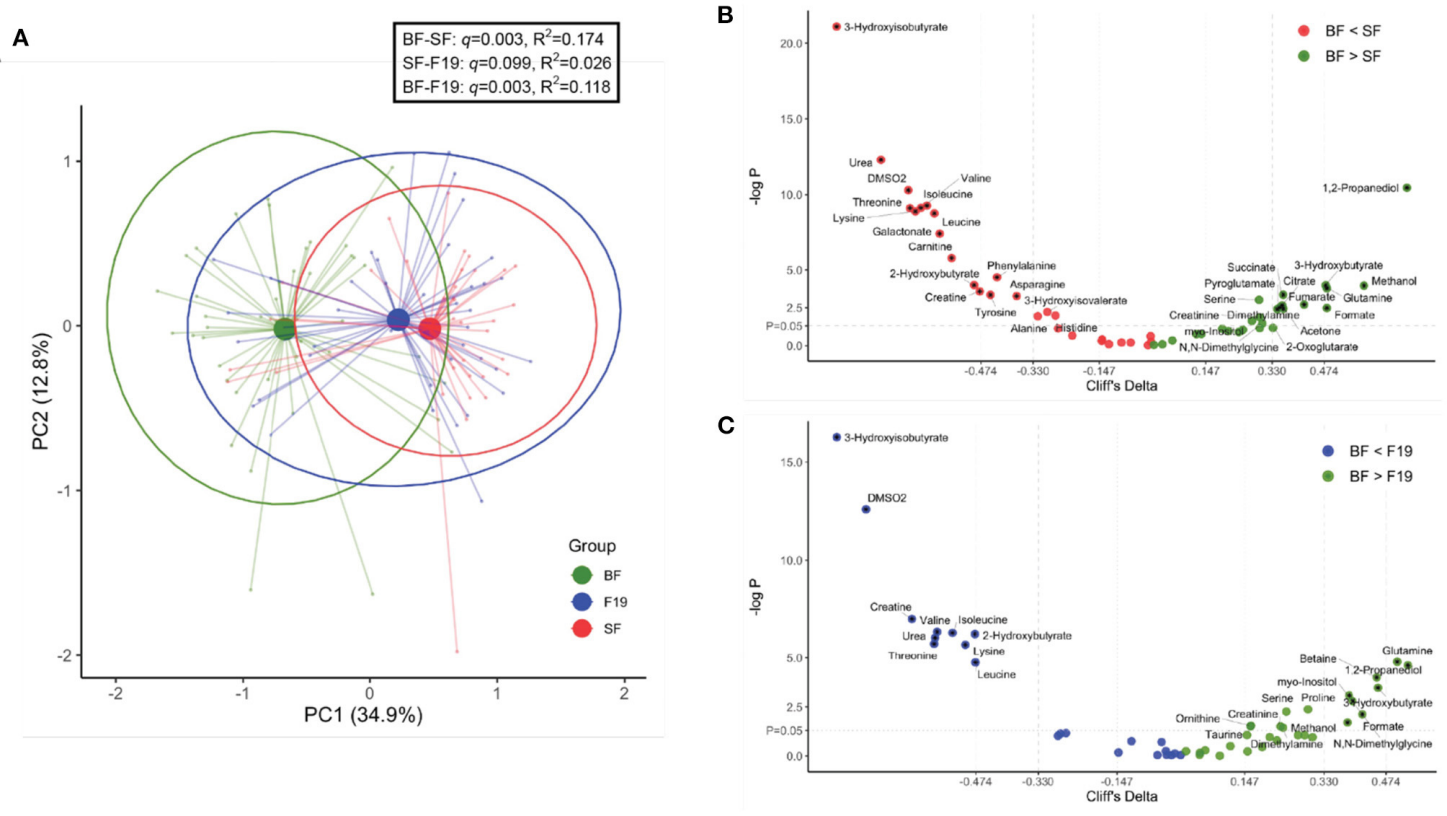
Comparison of the serum metabolome at 4 months of age of infants fed standard formula (SF; *n* = 40) or standard formula supplemented with *L. paracasei* F19 (F19; *n* = 41) and breast-fed infants (BF; *n* = 42). **(A)** Principal components analysis (PCA) of the generalized log-transformed serum metabolite concentration obtained from ^1^H NMR analysis. The *L. paracasei* F19 supplemented group is represented in blue, the standard formula group in red, and the breast-fed reference group in green. Results of Pairwise PERMANOVA are provided in the table insert. **(B)** Volcano plot showing Cliff's delta effect sizes vs. the log-transformed *p*-value comparing the standard formula group (red) and the breast-fed reference group (green). A *p*-value of 0.05 is indicated by the horizontal line. The vertical lines correspond to the cutoff between small and medium as well as medium and large effect sizes. Asterisks correspond to a *p*-value < 0.05 and at least a medium effect size after correcting for hospital. The color represents whether the concentration is higher in breast-fed infants (green) or infants fed standard formula (red). **(C)** Volcano plot showing Cliff's delta effect sizes versus the log-transformed *p*-value comparing the *L. paracasei* F19 supplemented group (blue) to the breast-fed reference group (green).

We have previously shown that in both the post-prandial and semi-fasting states, there are profound differences in the serum metabolome between BF and FF infants (4–7, 9, 11). In this study, as expected, the SF group differed from the BF reference group (Figures 2A,B), with metabolome differences similar to what was previously reported. Here, significantly higher levels of several amino acids (including valine, isoleucine, leucine, lysine, threonine, phenylalanine, asparagine, tyrosine, alanine, and histidine) and other metabolites [3-hydroxyisobutyrate, 2-hydroxybutyrate, 3-hydroxyisovalerate, creatine, carnitine, dimethyl sulfone (DMSO_2_), galactonate, and urea], and lower levels of 1,2-propanediol, ketone bodies (3-hydroxybutyrate and acetone), glutamine, methanol, formate, fumarate, citrate, succinate, pyroglutamate, serine, dimethylamine, creatinine, myo-inositol, 2-oxoglutarate, and N,N-dimethylglycine were observed in the serum of SF infants compared to BF infants (*p* < 0.05). Comparison of F19 infants to BF infants revealed a similar pattern in metabolites but with fewer significantly different metabolites and lower magnitudes of effect sizes than comparison of SF infants to BF infants (Figure 2C). Metabolites higher in F19 infants compared to BF infants included 3-hydroxyisobutyrate, DMSO_2_, creatine, urea, 2-hydroxybutyrate, and several amino acids (including valine, isoleucine, threonine, lysine, and leucine). Compared to BF infants, F19 infants exhibited lower levels of glutamine, 1,2-propanediol, 3-hydroxybutyrate, myo-inositol, serine, creatinine, formate, dimethylamine, N,N-dimethylglycine, betaine, proline, methanol, ornithine, and taurine.

**Figure 3:**
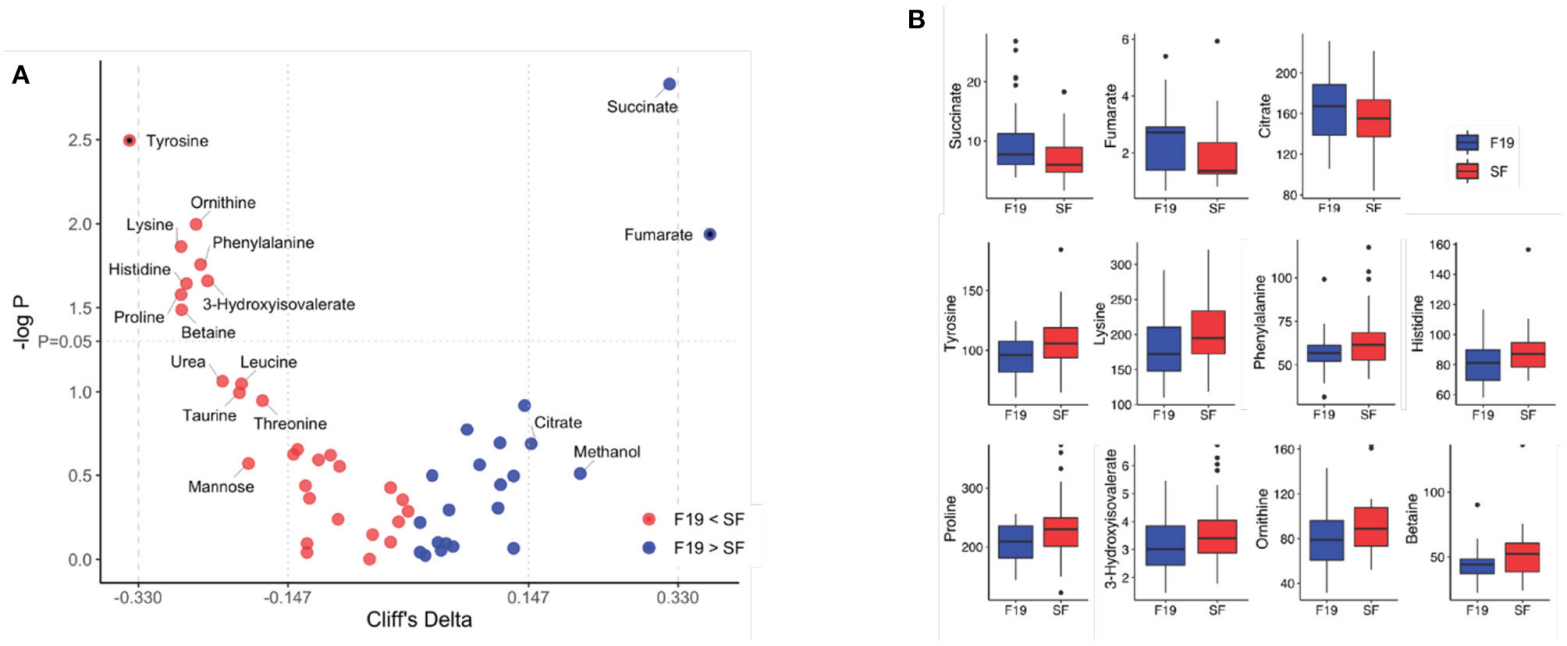
Comparison of the serum metabolome of infants fed standard formula with infants fed standard formula containing *L. paracasei* F19 at 4 months of age. **(A)** Volcano plot showing Cliff's delta effect sizes versus the log-transformed *p*-value comparing the standard formula group (red) and the *L. paracasei* F19 supplemented group (blue). A *p*-value of 0.05 is indicated by the horizontal line. The vertical lines correspond to the cutoff between small and medium as well as medium and large effect sizes. Asterisks correspond to a *p*-value < 0.05 and at least a medium effect size after correcting for hospital. The color represents whether the concentration is higher in infants fed standard formula (red) or infants provided the *L. paracasei* F19 probiotic (blue). **(B)** Box plots of metabolite concentrations in μmol/L comparing infants fed standard formula (red) with infants provided the *L. paracasei* F19 probiotic (blue).

Given that the F19 infants had a serum metabolome more similar to SF infants than to BF infants, we sought to determine the difference between the F19 and SF groups. Using a linear mixed-effect model, with hospital as a random effect, *p*-values and Cliff delta effect sizes were calculated and are summarized in a volcano plot (Figure 3A). Compared to the SF group, the F19 group had higher levels citric acid cycle intermediates (citrate, succinate, and fumarate) and methanol, and lower levels of several amino acids (tyrosine, lysine, phenylalanine, histidine, proline, leucine, and threonine), 3-hydroxyisovalerate, ornithine, betaine, urea, taurine, and mannose. The metabolites with at least a medium effect size and a *p* < 0.05 were summarized in Figure 3B. One of the branched chain amino acids, leucine, as well as taurine and urea were lower in F19 infants compared to SF infants with medium effect sizes, but these were not significant (*p* < 0.1).

Several SF (*n* = 6) and F19 (*n* = 12) infants had serum metabolomes consistent with a BF infant's metabolome, with substantial levels of 1,2-propanediol [a metabolite that is formed by fermentation of milk oligosaccharides by select strains of bifidobacteria that contain the genes to catabolize fucose (31–33)] and ketone bodies, with significantly lower levels of branched chain amino acids. Further, there were a few infants in the BF group that had levels of these metabolites that were consistent with FF infants (4–7, 9, 11). Removal of these samples from analysis resulted in no difference in our reported results suggesting that their presence in the dataset did not inadvertently skew the results.

## Discussion

Probiotics, including *Lactobacillus* and *Bifidobacterium*, have been studied as additives to infant formulas for many years [reviewed in (34–37)]; however, few studies have directly measured the metabolic impact. We previously studied the role of *Bifidobacterium animalis* subsp. *lactis* (*B. lactis*) supplementation on the rhesus infant metabolome and microbiome (4), and observed significant impacts on the fecal, urine, and serum metabolomes as well as microbiome compared to rhesus infants fed a standard formula. However, despite these changes, *B. lactis* supplementation did not override the impact of formula-feeding, nor pushed the metabolome or microbiome to a state more similar to BF infant (4).

In the current study, we assessed the impact of supplementation with *L. paracasei* strain F19 in human infants on the microbiome and the metabolome. As reported previously for this cohort (29, 30), no adverse effects of the F19 probiotic were observed in infants when the probiotic was consumed with a standard infant formula; however, a significant cytokine response at 4 months of age [higher IL-2 in the F19 group relative to BF, and lower IFN-γ in the F19 group relative to the SF group (with no difference to BF)] was observed (30). The reason for this difference could be due to the probiotic itself, the probiotic-modulated gut microbiota, and/or the probiotic and microbiota-associated metabolites that interact with immune cells and epithelial cells. Indeed, it is known that gut microbiota can alter immune responses (38–46).

As expected, at 4 months (the end of the supplementation period), the SF and F19 infants had a similar microbial community structure (besides the higher relative abundance of *Lactobacillus* in the F19 group), which was significantly different from BF infants. We have observed this in other studies (3, 4, 6, 7), and this shows that there is a distinct microbial composition for BF infants compared to FF infants. As we have also shown in previous studies (3, 6), at 12 months, infants from all three groups were indistinguishable from one another, which suggests that current diet, rather than previous diet, impacts the microbial composition measured through 16S sequencing.

Prior to the start of the study (at baseline), the microbial community structure of infants recruited to the F19 group was significantly different from the BF reference group. One reason could be the higher rate of Caesarian section births (47% in the F19 vs. 35% in the BF group); however, the SF group had more Caesarian section births (68%) than the F19 group, so it is unlikely that this was a contributing factor. A more likely explanation is that infants in the FF groups were already receiving formula and possibly more infants in the F19 group had a greater consumption of formula at this age than those in in the SF group, but the amount of formula consumption at recruitment was not recorded. Nonetheless, at baseline, the Evenness Index and Shannon Index were similar for the F19 and SF group, and higher than the BF reference group (Figure 1D).

Interestingly, after the intervention, microbial community diversity in the F19 group was significantly lower than in the SF group, and comparable to the BF group. Whether community diversity is associated with its stability or instability is controversial (47), but lower microbial diversity over the first few months of life has been consistently associated with breastfeeding (48). Human milk contains selective growth substrates for specific microbes (i.e., human milk oligosaccharides) that have the additional property of inhibiting growth of undesirable bacteria (49). It is not known whether it is the HMOs, the *Bifidobacterium*, or a combination of the two that are responsible for limiting the type and number of organisms colonizing the infant gut, but it would appear in this case that *L. paracasei* F19 has the ability to alter the evenness (but not the richness) of commensal bacteria. This makes sense as the overall composition would be dictated by the diet (i.e., formula-fed), but the relative abundance of each species could be modulated by *L. paracasei* F19. This may occur through the production of antimicrobial compounds including lactic acid and bacteriocins from *L. paracasei* F19, or could arise due to competition for nutrients (50). It is also interesting that although the relative abundance of Firmicutes was higher in the F19 group compared with the BF group, it was not higher than the SF group despite supplementation with the probiotic. Remarkably, supplementation with F19 tended to increase the average relative abundance of Actinobacteria, specifically the genus *Bifidobacterium*, in the F19 group to levels similar to the BF group, although it is unlikely that there are similar species / strains of *Bifidobacterium* in the BF and F19 groups since HMOs were not present in the formula. To understand if F19 has an ecological relationship with species of *Bifidobacterium* in the gut of formula-fed infants, future analyses of bacterial species and strains or metagenomics should be performed. Nonetheless, this is an important observation as bifidobacterial species in general have been shown to have many health benefits, including immunomodulatory effects (41–43), which could together with *L. paracasei* F19 drive some of the changes in the levels of IL-2 and/or IFN-γ reported previously in this cohort (30). It is interesting to note that similar observations were not observed in an infant cohort provided *L. paracasei* F19 as part of weaning foods (28, 51), thus the changes observed here may be specific to early supplementation.

Serum metabolites provide a reflection of recent food intake as well as information regarding metabolic status. Supplementation with *L. paracasei* F19 resulted in differences in the serum metabolome when compared with SF fed infants and suggests that F19 supplementation may mitigate reported metabolic discrepancies between BF and FF infants (9, 11). Specifically, BF infants compared to FF infants exhibited observed lower levels of serum amino acids and higher levels of citric acid cycle intermediates. Here, F19 supplementation lowered the median concentrations of several essential amino acids (tyrosine, lysine, leucine, phenylalanine, histidine) and 3-hydroxyisovalerate, and increased levels of citrate, succinate, and fumarate in the serum of FF infants, resulting in concentrations comparable to the BF group. The median concentrations of ornithine and betaine were similar in the SF and BF groups, but were lower in the F19 group. Changes in plasma amino acid concentrations with *L. paracasei* probiotic supplementation have been reported (52). Specifically, consumption of plant protein with an *L. paracasei* probiotic increased several amino acids in the blood, which was interpreted as changes in digestion of the plant protein (52). In a humanized mouse model, metabolic changes were observed in liver, plasma, fecal and urine when mice were provided an *L. paracasei* supplement compared with control (53). Supplementation with *L. paracasei* F19 in infant formula may modulate protein digestion / utilization and/or amino acid metabolism, as observed with reduced levels of some amino acids and nitrogen waste products in serum. This trait is a metabolic characteristic of BF infants that may benefit insulin sensitivity and metabolic function later in life (5, 7, 9–11) and whether this would yield any long-term consequence needs further investigation. Although not analyzed in this study, the fecal metabolome or metagenomics could provide more insights on how bacterial metabolism is interrelated with the host metabolome with *L. paracasei* F19 supplementation.

A limitation of the current study includes a lack of dietary records of potential other foods consumed by infants (other than breast milk or study formulas), although it was recommended for parents not to provide other foods during the intervention. In conclusion, our study provides insights on how probiotic supplementation with *L. paracasei* F19 induces changes in host nutrient utilization and energy metabolism, characterized by increased citric acid cycle metabolites and reduced protein degradation products, suggesting a shift in metabolism closer to BF infants. While the *L. paracasei* F19 supplementation significantly increased the relative abundance of *Lactobacillus*, it did not induce significant compositional changes in the other bacteria compared to the infants consuming the standard formula. However, there was a noticeable change in the microbial diversity and the tendency of harboring more *Bifidobacterium* in the gut. To date, the clinical outcomes of this observation are not known, and the long-term effects need to be assessed in future studies.

## Materials and Methods

### Subjects

This clinical trial was a multicenter double-blind, randomized controlled trial with infants from 21 ± 7 days until the end of the 4^th^ month of age. Infants were followed until the age of 1 year. The design and clinical results of the study were previously published (29). The trial was approved by the Institutional Review Board (IRB) at the University of California Davis and the regional Ethical Review Boards in Nanjing, Shanghai, and Beijing in China (ClinicalTrials.gov Identifier: NCT01755481). Inclusion criteria encompassed healthy infants born full term with a birthweight between 2,500 g and 4,000 g. For infants in the breast-fed reference group (BF), inclusion criteria also included exclusive breastfeeding from birth, with mothers intending to breast-feed for at least 5 months and providing at least 80% of the calories to their infants from breast milk. Additional inclusion criteria for the two formula groups (FF) included infants of mothers who either could not breast-feed, or voluntarily resigned from breast-feeding by 28 days.

Except for the addition of the probiotic *Lactobacillus paracasei* ssp. *paracasei* strain F19 to one of the formulas [at a dose of 10^8^ CFU/L; 8.33 × 10^7^ CFU/day based on an average formula consumption of 833 mL/day (29)] (F19), the composition of the study formulas was exactly the same. The formulas were manufactured in Hohot, China using a bovine milk powder provided by Arla Foods amba, Denmark, and the *L*. *paracasei* ssp. *paracasei* strain F19 provided by Chr. Hansen, Denmark. The nutrient composition of the formula was previously published (29).

Prior to the intervention, infants in the FF groups were provided standard formula (SF) if formula feeding had been initiated. Between the 5^th^ and 6^th^ month of life, infants in both FF groups were provided SF. For BF infants, if milk supply was insufficient, SF was used, but was not to exceed 20% of total calorie intake based on a monthly three-day intake record (29). Although feeding of other foods or formulas was not recommended during the intervention, data on consumption of other foods or formulas was not recorded. Parents were recommended to introduce complementary foods to the infants no later than 6.5 months of age. The group code was blinded from study staff and enrolled participants, and was not broken until sample extraction, lab analysis, and generation of concentration/relative abundance data had been completed.

To confirm consumption of *L*. *paracasei* ssp. *paracasei* strain F19 in the F19 group, the amount in the stool was determined using quantitative PCR using the same primers and conditions as described by Sieuwerts and Håkansson (54).

### Serum Metabolomics Analysis

A total of 150 subjects (*n* = 50 per group) were randomly chosen from 179 BF, 167 SF and 167 F19 infants completing the study (29), and serum samples collected from those infants at 4 months of age were used for metabolomics analysis. Out of the potential 150 samples, 6 samples had too small (< 50 μl) or no volume, 15 samples were hemolyzed (identified by a bright red color), 3 samples from the F19 group were from infants with no detectable levels of *L*. *paracasei* ssp. *paracasei* strain F19 in their stool, and 3 samples had noisy NMR spectra or potential contamination. Exclusion of these samples left 123 samples (*n* = 42 BF, *n* = 40 SF, and *n* = 41 F19) for metabolomics analysis.

Serum samples were prepared by filtering through 3,000 MW cutoff Amicon filters (Merck Millipore, MA, USA) followed by the addition of potassium phosphate buffer (pH 6.7) and an internal standard containing 5 mM 3-(trimethylsilyl)-1-propanesulfonic acid–d6 (DSS-d6) and 0.2% NaN_3_ in 99.8% D_2_O as previously described (6). NMR spectra were acquired on a Bruker Avance 600 MHz NMR spectrometer at 25°C using the Bruker noesypr1d experiment as previously described (7). NMR spectra were processed using Chenomx NMR Suite v8.2 Processor (Chenomx Inc., Edmonton, Canada) (RRID:SCR_014682), and metabolites were identified and quantified using Chenomx NMR Suite Profiler v8.2 based on the internal standard DSS-d6 as described (55). Quantified metabolite concentrations were corrected for dilution and are expressed as absolute concentrations in μmol/L.

### Fecal Microbiome Sequencing

One hundred and fifty-one subjects (*n* = 51 BF, *n* = 50 SF and *n* = 50 F19) from the original cohort were randomly chosen for 16s rRNA gene sequencing, and a total of 453 fecal samples representing three time points [baseline (21±7 d), end of the 4^th^ month and 12 months] from 151 infants were used. Fecal samples were collected by a parent or guardian from the infant's diaper, and placed in a provided container, which was placed in a freezer bag and stored at −20°C in a home freezer until the hospital visit (29). At the hospital, samples were placed in a −20°C freezer and stored until transport on dry ice to the Netherlands Organization for Applied Scientific Research (TNO) for analysis.

DNA from fecal samples was isolated and V4 hypervariable region of the bacterial 16S rRNA gene was amplified with universal bacterial 515F−806R primer pair as described (6). Prepared libraries were sequenced on the paired-end 2 × 200 bases Illumina MiSeq platform with a MiSeq Reagent Kit v2 (MS-102-2003, Illumina).

Paired sequence reads from 453 selected samples (BF *n* = 51, SF *n* = 50, F19 *n* = 50) and 48 quality control samples were pre-processed in QIIME2 (version 2019.4; https://qiime2.org/) as described (6). The following subject's samples were excluded from sequencing analyses: (1) one subject in the F19 group identified as quantitative-PCR negative for the F19 strain at 4 months; (2) 13 non-exclusive breastfed infants in the BF group; and (3) infants treated with antibiotics during the intervention (3 BF, 9 SF, and 6 F19), leaving a total of 37 BF, 43 F19, and 41 SF. Raw sequence and processed files have been deposited through QIITA (study ID: 12874) in the European Nucleotide Archive (ENA) at EMBL-EBI under accession number PRJEB38295 (https://www.ebi.ac.uk/ena/browser/view/PRJEB38295).

### Statistical Analysis

To determine associations in the microbiota with diet, principal coordinate analysis (PCoA) was performed. Comparisons for beta diversity metric estimates were made by computing pairwise Permutational Multivariate Analysis of Variance (PERMANOVA) (56) based on 999 permutations. Kruskal-Wallis tests were used for comparing alpha diversity estimates between categorical variables. Longitudinal analyses were performed on changes in alpha diversity estimates over time using paired-difference testing with the q2-longitudinal plugin in QIIME2, followed by visualization using qiime2R (ver. 0.99.1) and ggplot2 (ver. 3.1.1) packages in R. Taxonomic assignments of representative sequences were conducted for 515F/806R primer pair through a naïve-Bayes classifier trained on the Greengenes ver. 13_8 database at 99% OTU similarities. Differential abundance analysis of each taxon was determined using ANCOM (Analysis of composition of microbiomes) (57). Taxa bar plots were generated using vegan (ver. 2.5-5) and ggplot2 packages in R.

For metabolomics, statistical computation was performed in R (ver. 3.5.3.) and visualized using the *ggplot2* package unless stated otherwise. Principal component analysis (PCA) (*prcomp* function; mean-centered, non-scaled, and log-transformed data) was used to visualize the data. Groups were compared using pairwise PERMANOVA on Euclidean distances. Two-way Analysis of Variance (ANOVA, *aov* function; log-transformed data) were computed to test for significant effects of diet, sex, region, hospital, and mode of delivery as previously described (6). The magnitude of the diet effect on the metabolome was estimated by calculating Cliff's Delta effect size estimates (*cliff.delta* function).

## Data Availability Statement

The original contributions presented in the study are publicly available. The microbiome data can be found at: ENA, PRJEB38295 (https://www.ebi.ac.uk/ena/browser/view/PRJEB38295?show=reads). The metabolome data supporting the conclusions of this article will be made available on request by the authors, without undue reservation.

## Ethics Statement

The studies involving human participants were reviewed and approved by Institutional Review Board (IRB) at the University of California Davis and the Regional Ethical Review Boards in Nanjing, Shanghai, and Beijing in China (ClinicalTrials.gov Identifier: NCT01755481). Written informed consent to participate in this study was provided by the participants' legal guardian/next of kin.

## Author Contributions

ZL, BC, YP, XL, OH, and BL designed and ZL, YP, and XL performed the clinical trial. HL analyzed samples for metabolomics and performed all metabolome, microbiota, and subject data analyses. Manuscript written by HL and CS. OH and BL edited, and all authors approved the final manuscript.

## Funding

The authors declare that this study received funding from Arla Foods amba. The funder was involved in the study design, but not in the conduct of the study, collection of samples, analysis or interpretation of the data, nor in the writing of this article or the decision to submit it for publication. This work was also supported by the USDA National Institute of Food and Agriculture Hatch project 1021411, and the Kinsella Endowed Chair in Food, Nutrition, and Health (to CMS). The 600 MHz NMR is supported through NIH grant 1S10RR011973-01.

## Conflict of Interest

BC is employed by Arla Foods amba. BL and OH have served as consultants for Arla Foods amba. The remaining authors declare that the research was conducted in the absence of any commercial or financial relationships that could be construed as a potential conflict of interest.

## Publisher's Note

All claims expressed in this article are solely those of the authors and do not necessarily represent those of their affiliated organizations, or those of the publisher, the editors and the reviewers. Any product that may be evaluated in this article, or claim that may be made by its manufacturer, is not guaranteed or endorsed by the publisher.
